# Faster RCNN‐based detection of cervical spinal cord injury and disc degeneration

**DOI:** 10.1002/acm2.13001

**Published:** 2020-08-14

**Authors:** Shaolong Ma, Yang Huang, Xiangjiu Che, Rui Gu

**Affiliations:** ^1^ Department of orthopedics China‐Japan Union Hospital of Jilin University Changchun, Jilin China; ^2^ College of Computer Science and Technology Jilin university Changchun China

**Keywords:** cervical spinal cord injury, convolutional neural networks, disc degeneration diseases, faster R‐CNN, MRI

## Abstract

Magnetic resonance imaging (MRI) can indirectly reflect microscopic changes in lesions on the spinal cord; however, the application of deep learning to MRI to classify and detect lesions for cervical spinal cord diseases has not been sufficiently explored. In this study, we implemented a deep neural network for MRI to detect lesions caused by cervical diseases. We retrospectively reviewed the MRI of 1,500 patients irrespective of whether they had cervical diseases. The patients were treated in our hospital from January 2013 to December 2018. We randomly divided the MRI data into three groups of datasets: disc group (800 datasets), injured group (200 datasets), and normal group (500 datasets). We designed the relevant parameters and used a faster‐region convolutional neural network (Faster R‐CNN) combined with a backbone convolutional feature extractor using the ResNet‐50 and VGG‐16 networks, to detect lesions during MRI. Experimental results showed that the prediction accuracy and speed of Faster R‐CNN with ResNet‐50 and VGG‐16 in detecting and recognizing lesions from a cervical spinal cord MRI were satisfactory. The mean average precisions (mAPs) for Faster R‐CNN with ResNet‐50 and VGG‐16 were 88.6 and 72.3%, respectively, and the testing times was 0.22 and 0.24 s/image, respectively. Faster R‐CNN can identify and detect lesions from cervical MRIs. To some extent, it may aid radiologists and spine surgeons in their diagnoses. The results of our study can provide motivation for future research to combine medical imaging and deep learning.

**Abbreviations**
CNN: convolutional neural networks
Conv: convolutional
CSCI: cervical spinal cord injury
DDD: disc degeneration diseases
Faster R‐CNN: faster‐region convolutional neural networks
FCN: fully convolutional network
FS: fat saturation
mAP: mean average precision
MND: motor neuron disease
MRI: magnetic resonance imaging
NMS: Non‐maximum suppression
PACS: picture archiving and communication systems
ResNet: residual network
ROI: region of interest
RPN: region proposal network
STIR: short tau inversion recovery
T1WI: T1‐weighted Image
T2WI: T2‐weighted Image

## 1 INTRODUCTION

Traumatic diseases have become common with the advancements in modern society. In general, severe neurologic deficits due to injuries, including intramedullary hematoma and spinal cord contusion associated with edema encompassing the spinal cord,^1^ can be observed in magnetic resonance imaging (MRI) signals. Many studies have shown that MRI can detect physiological and morphological changes (such as swelling and asymmetry) based on variations in water molecules by measuring alterations in the intensity of tissue signals.^2^ Furthermore, damages caused by cervical disc degenerative diseases (DDD) and traumatic spinal cord injury (SCI) can be confirmed by MRI, which is the basis for identifying spinal cord diseases and neurological recovery.^3^, ^4^ Therefore, diagnosis is a key step to treatment and controlling lesions on soft tissue, and MRI could provide better diagnostic tools when there is uncertainty regarding diagnosis.^5^, ^6^

Recently, deep learning‐based models, especially convolutional neural network (CNN) models, have been efficient in object detection. Convolutional neural network models have been applied in several medical disciplines, including radiology,^7^, ^8^ pathology,^9^ dermatology,^10^ and ophthalmology.^11^ Previous studies focused primarily on brain diseases compared to diseases of the spinal cord because MRI has been successful in diagnosing brain‐related illness. In addition, spinal cord diseases exhibit more variations in their morphology and signals in sagittal MRI.^12^, ^13^, ^14^, ^15^ Only a few studies have investigated spinal cord diseases on MRI using CNN models. Gros et al. conducted a study that utilized a sequence of two CNNs to segment the spinal cord and/or intramedullary multiple sclerosis lesions based on a multi‐site clinical dataset, and their segmentation methods showed a better result compared to previous CNN models.^16^ However, the spinal cord diseases that they studied did not have specific locations and usually occurred in multiple areas, such as the brain, cerebellum, and lateral ventricles. In addition, MRI data on cervical diseases are insufficient, which has frustrated researchers in object detection/segmentation. As is known, DDD and SCI are the most common diseases of the cervical spine in clinical medicine, and sagittal MRI is increasingly being recognized for its contribution in assessing disease severity in patients with SCI and DDD.^17^, ^18^ However, the classification and detection of lesions for DDD and SCI on MRI images on the basis of deep neural networks appear to be limited as published studies in this regard are lacking in the literature. In addition, the performance of traditional deep learning methods are unsatisfactory, and traditional CNN have several defects.^8^, ^19^, ^20^ To address this, some novel algorithms capable of powerful processing in object detection have been proposed; an example of such an algorithm is Faster R‐CNN,^19^ which offers advantages in terms of accuracy and detection speed. Therefore, we investigated the feasibility of using faster‐region convolutional neural networks (Faster R‐CNN^19^) with a combination of the pre‐trained VGG/Resnet^21^ (to extract features) to identify and detect spinal cord diseases on the MRI dataset used in this study. Experimental results show that this method has good recognition performance.

## 2 MATERIALS AND METHODS

### 2.A Data collection

Patients with cervical diseases were admitted to our hospital between January 2013 and December 2018. Two diseases were considered as inclusion criteria: cervical DDD and traumatic SCI patients, which mainly refer to cervical disc herniation and changes in spinal cord signal due to injury, respectively. Simultaneously, spinal cord tumors, syringomyelia, motor neuron disease (MND), and peripheral polyneuritis were used as exclusion criteria.

Patients were subjected to a cervical spine MRI performed by radiologists using surface‐coil MRI with 1.5 or 3.0 T. The MRI included T1‐weighted image (T1WI), T2‐weighted image (T2WI), and short tau inversion recovery (STIR) or fat saturation (FS); STIR and FS can be regarded as one type.^22^, ^23^ In clinical procedures, MRI usually includes three types of images — T1WI, T2WI, FS, or STIR images; the typical changes observed in tissue during MRI are listed in Table 1. Based on the results from images and disease incidence, a total of 1000 patients were enrolled from the picture archiving and communication systems (PACS) station, including 690 men and 310 women. In addition, data of 500 people who were diagnosed as negative were collected (without DDD and SCI) to obtain better real‐time training results. The patients were divided into three groups: “normal group,” “disc group,” and “injured group.” Finally, all the images were desensitized before being used (e.g., removal of name, age, date of examination, and sex).

**Table 1:** Characteristics of tissues in magnetic resonance imaging.

Tissues signal shown in MRI			
Type	T1WI	T2WI	STIR/FS
Water	Hypo‐intense	Hyper‐intense	Hyper‐intense
Fat	Hyper‐intense	Hyper‐intense	Hyper‐intense
Calcification	Hypo‐intense	Hypo‐intense	Hypo‐intense
Ossification	Hypo‐intense	Hypo‐intense	Hypo‐intense
Gasification	Hypo‐intense	Hypo‐intense	Hypo‐intense
Bleeding (3d‐2w)	Hyper‐intense	Hyper‐intense	Hypo‐intense

### 2.B Data preparation

**Fig. 1:**
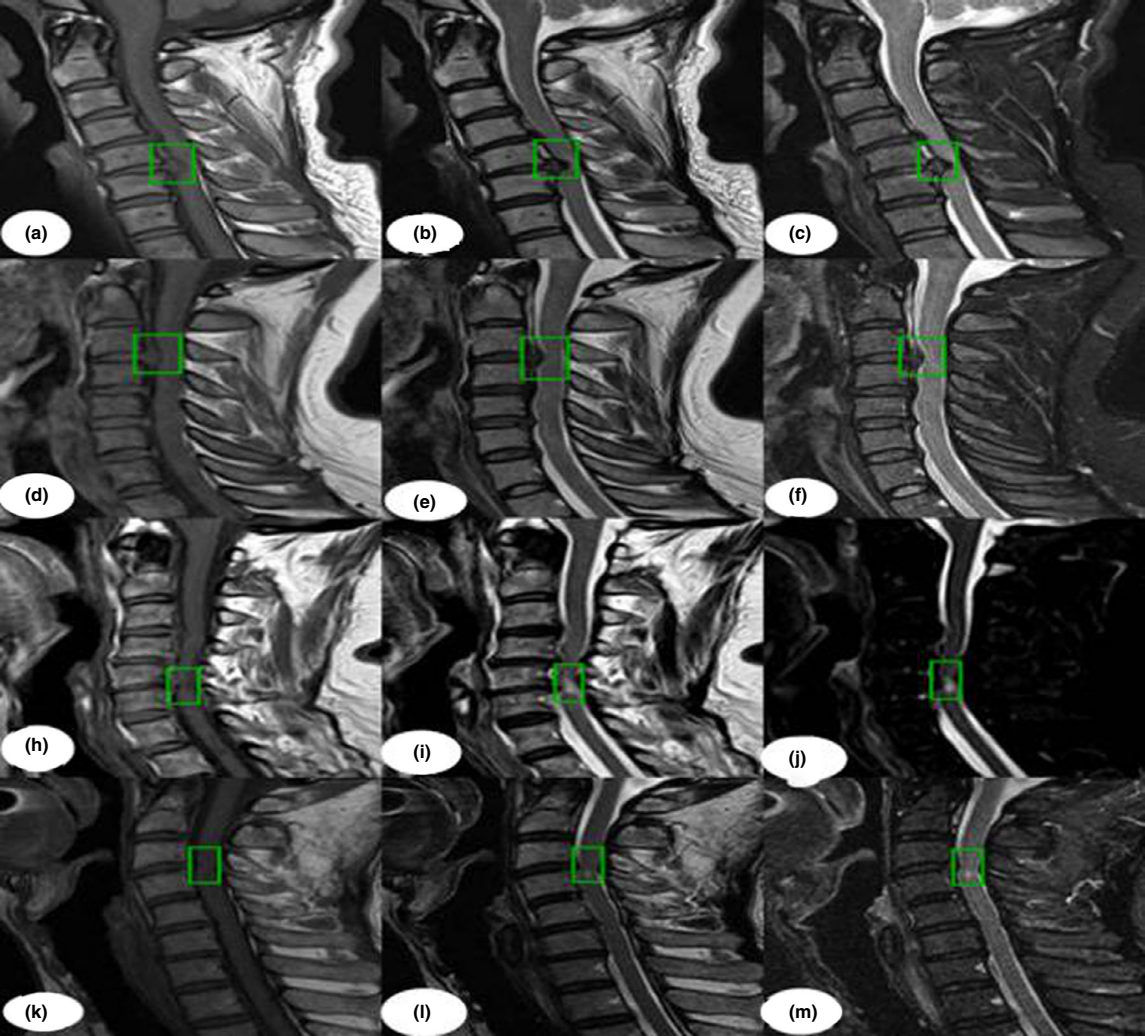
Lesions on magnetic resonance imaging (MRI) annotated in a bounding box by two spine surgeons. Images a–f show the typical T1WI, T2W2, and STIR as "disc groups" labeled with the region of interest (ROI); images h–m show examples of the "injured group" marked with the ROI. The dataset is also naturally imbalanced with respect to the lesion classes, and the "disc group" clearly dominates with 80% of the total images in our training dataset.

**Fig. 2:**
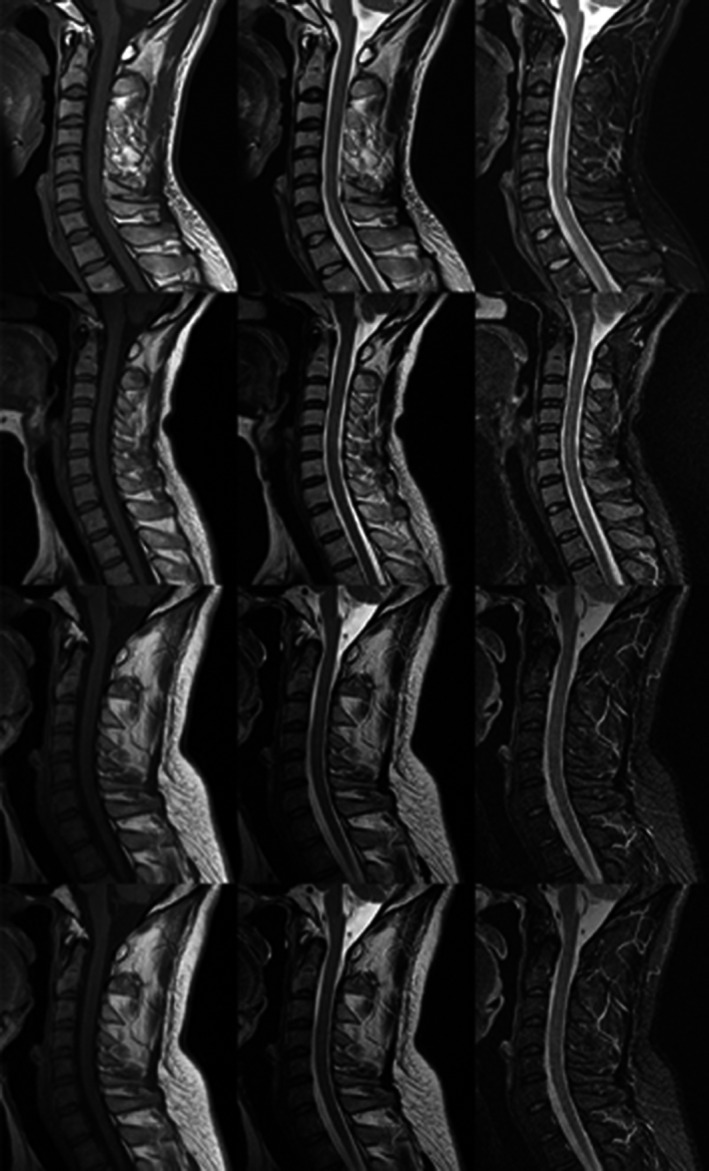
Comparison between normal magnetic resonance imaging (MRI) and abnormal MRIs.

The dataset was randomly split into two parts: 1200 (80%) patients for training and 300 (20%) patients for validation; this was done to simulate the proportion of the incidence in reality. Additionally, 500 MRI images were classified as a testing set to demonstrate detection performance, and the number of images in the “normal group,” “disc group,” and “injured group” were 200, 200, and 100, respectively. The training and validation sets used a bounding box to show the location of the lesion, as shown in Fig. 1, while the “normal group” without a bounding box is shown in Fig. 2. In this process, two experienced spine surgeons labeled the bounding boxes using LabelMe Tool box‐master. Before feeding the dataset into the network, we cropped the center to eliminate differences from raw data, which was generated from the PACS station. Finally, the number of dataset images was increased by a factor of 10 after horizontal flip and contrast enhancement.

### 2.C Overview of the study

In clinical diagnosis, the commonly used weighted images of cervical MRI are T1WI, T2WI, and FS. Doctors need to combine the information from three different images to comprehensively judge the patient's condition.^5^, ^6^ Typically, in medical classification and detection problems, the three types of images are used as independent inputs. These weighted images are considered as a data augmentation method. Other methods integrate these three single‐channel images at the third dimension, and regard it as a three‐channel image. However, these methods have some problems. The former will cause the same patient to be diagnosed with different diseases by inputting different weighted images, resulting in confusing results. The latter uses the combination of three single‐channel images to simulate the input of a three‐channel color image. However, because the distribution of pixel values of the synthetic image is not consistent with the distribution of pixel values from the large dataset of real images, like ImageNet, the efficiency of the network will be reduced when using the pre‐trained model, and the network cannot be pre‐trained with other medical datasets.

In this dataset, each image is divided into three categories: normal, SCI, and DDD. SCI and DDD signals do not appear on any image at the same time, which means that the classification information of the three images is the same for a patient, although the location of the bounding box obtained by the three images on the network may be slightly different. Therefore, integrating the classification information of the three images becomes a key problem in the design of the network structure.

**Fig. 3:**
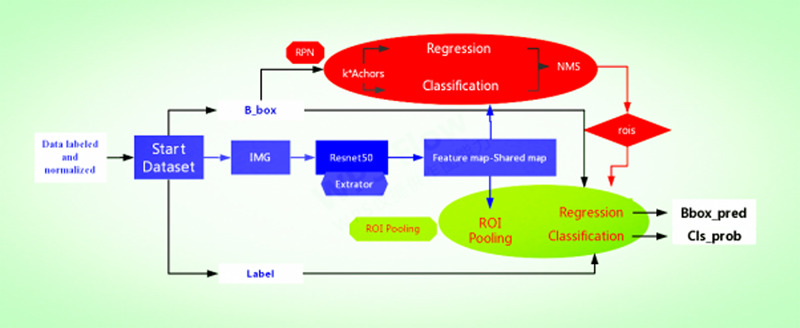
Overview of the traditional Faster R‐convolutional neural network framework based on our method, showing each sub‐network.

We used Faster R‐CNN as the main structure of the network. Currently, Faster R‐CNN is the most popular two‐stage detection network, and it is used in many medical image detection problems.^22^, ^23^ The Faster R‐CNN mainly includes a feature extractor, region proposal network (RPN), RoI pooling, and classifier. Primarily, Faster R‐CNN consists of two parts. One is the RPN; it is a fully convolutional network (FCN) for generating object proposals that will be fed into the second module. The second is the Fast R‐CNN^24^ detector whose purpose is to refine the proposals and the sketch map of detection processing, as shown in Fig. 3. It should be highlighted that RPN and Fast R‐CNN share the convolutional layers in order to save time.^19^ Specifically, RPN is an FCN that simultaneously predicts object bounds and objectness scores per image and generates high‐quality region proposals, which are used by Fast R‐CNN for detection. Then, the region of interest (ROI) pooling layer takes ROIs and convolutional features as inputs and generates the bounding box of the objects as well as the corresponding class name as the outputs, which contain cls_score and b_box pred. Based on the trade‐off between network complexity and performance, VGG/Resnet‐50 is used as the backbone network in this paper; that is, the part before VGG/Resnet‐50, the global average pooling and the full connection layer, is used as the feature extractor for Faster R‐CNN.

**Fig. 4:**
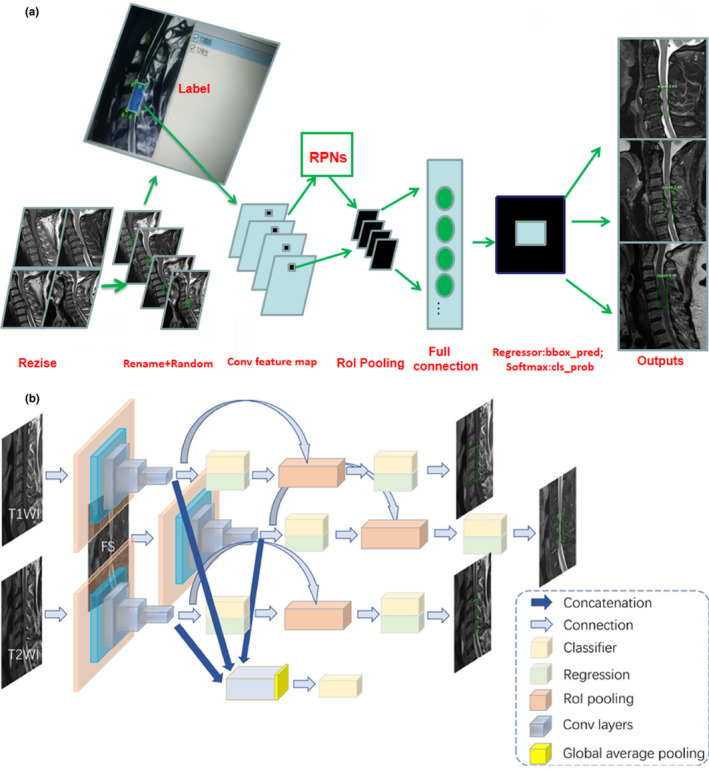
(a) Example of detection using processing Faster R‐convolutional neural network (R‐CNN) in magnetic resonance imaging. (b) Example of detection using Faster R‐CNN from each sub‐network.

**Fig. 5:**
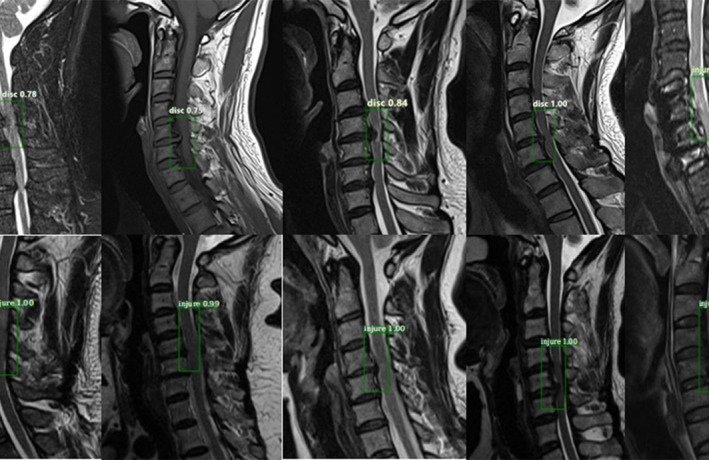
Example of detection results: from the outputs, each picture is surrounded by a target area and the corresponding classification name and predicted probability value. This is an efficient method to recognize the effects of lesions on magnetic resonance imaging images. The experimental results are observed to have reached the expected goal.

We present the Faster R‐CNN training the ResNet‐50 to illustrate the detection of lesions on MRI as an example. There are two steps: first, we combine the data according to the triples (triad patterns) as an input, that is, each input group includes the T1WI, T2WI, and FS images of one patient, which are recorded as (T1, T2, and FS). Each image in the triplet will be input into a separate network, respectively; more specifically, CNN inspired from ResNet‐50 of Faster R‐CNN was employed to detect the lesion on the T1, T2, and FS images. After extracting the bounding boxes from the images, we retain one or zero bounding box for judging per image, which is based on the confidence of the bounding box. In this work, RPN and the features at conv_4 layer are used to predict class‐specific box proposals. We set two scales (32, 64) and two aspect ratios (1:1, 1:2). We raised our threshold from 0.7 to 0.8 because many patients have minor and occult injuries that are not considered as lesions on the spinal cord. Then, we used the top‐ranked proposal region for detection after non‐maximum suppression (NMS)^25^; more specifically, we retained a maximum of one bounding box per image because for an SCI, a patient usually has only one specific lesion area existing on the MRI according to clinical diagnosis. To consistently classify these three networks for the three images, we add a synergic classifier after the feature extractor of each network, as shown in Fig. 4. To construct a synergic classifier, the feature map obtained from the last convolution layer of each feature extractor is combined by concatenation. In this paper, the combined size is 70 × 70 × 2048 × 3. After the global average pooling, the data are flattened and input into the full connection layer. Finally, the result is obtained by using the Softmax classifier. The addition of synergic classifiers groups the three networks into a whole network, and the three networks become the sub‐networks of the whole network. Because each sub‐network can generate a bounding box after the NMS, we use the NMS again for the “bounding” boxes of the three sub‐networks, and only retain the “bounding” box with the highest classification confidence. Finally, we use the bounding box obtained by the three sub‐networks to get the non‐maximum suppression again, and only retain the bounding box with the highest classification confidence. The result of Faster R‐CNN with VGG‐16/ResNet‐50 for detection lesion associated with cervical diseases on MRI after testing is presented in Table 2, and Fig. 5 shows some examples of the study.

**Table 2:** Recognition performance in this network.

Methods	Network	mAP (%)	Test time speed (s/image)
Faster R‐CNN	Resnet‐50	88.6	0.22
Faster R‐CNN	VGG‐16	72.3	0.24

mAP, mean average precision.

## 3 RESULTS

**Fig. 6:**
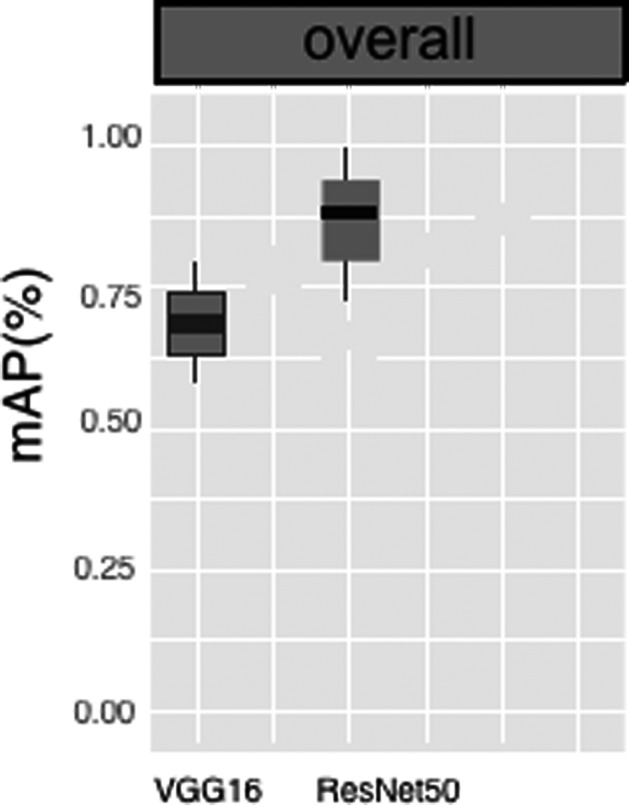
Histogram of the prediction results for the VGG16/Resnet 50 models. The sample sizes for the training set, validation set, and testing set are 3600, 900, and 500, respectively.

First, the datasets were desensitized, and scales were normalized and labeled. Examples of the imaging studies are illustrated in Figs. 1, 2, 3, 4. Next, to comprehensively evaluate the proposed detection system, the testing set was also collected from the PACS system to assure data uniformity; a sketch map of training detection processing is shown in Fig. 4. Furthermore, the mean average precision (mAP^26^) of the detection and the detection time were calculated; these are summarized in Table 2. From Table 1, it is clear that the architecture of Faster R‐CNN combined with ResNet‐50 and VGG‐16 network had good recognition of the lesion on cervical spinal cord MRI. The mAP was 88.6% and 72.3%, respectively, and testing time was 2.2 and 2.4 s/image, respectively. Figure 5 illustrates several examples of visualization images（including disc herniation and spinal cord injure）from the testing dataset through our model. Figure 6 shows the prediction results for the VGG16/Resnet 50 models.In the obtained images, apart from the frame, the corresponding damage probability also marks the image damage area.

## 4 DISCUSSION

MRI findings linked with abnormal conditions of the spinal cord can indirectly reflect several microscopic changes in lesions caused by DDD and SCI diseases in clinical settings.^27^ Our objective was to classify and localize the lesion's area on MRI based on Faster R‐CNN architecture with two kinds of CNN models (VGG/ResNet). mAP, the visualized images, and corresponding location information tables are acquired from the testing subjects in our method. The results show that Faster R‐CNN combined with VGG16 and ResNet‐50 as a backbone can predict the lesions on MRI, and Faster R‐CNN with ResNet‐50 has better prediction and speed for detecting lesions associated with cervical diseases on MRI after testing.

On the one hand, in the field of image recognition and visual learning tasks, CNNs in particular have rapidly become a methodology of choice for analyzing medical images because of their unique characteristic of preserving local image relations while performing dimensionality reduction,^27^, ^28^ such as residual network (ResNet). It not only allows the gradient to pass the shortcut to alleviate gradient disappearance but also allows the model to learn an identity function that ensures the performance of the higher layers to be as good as the underlying layers, if not worse.^21^ The main structure diagram of ResNet is shown in the study published by He et al.^29^ *Schmidhuber*, in his study,^30^ described the origin, operation process, development, and application of deep learning. On the other hand, Faster R‐CNN, as a novel algorithm, has been proposed with powerful processing speeds in object detection since 2015. To date, Faster R‐CNN has several advantages in regard to accuracy and detection speed. We found that the proposed method achieved effective recognition performance compared to VGG16, which is similar to the findings reported by Jung et al.^31^ In addition, the numerical experiments showed that Faster R‐CNN combined with novel CNN models has a better recognition performance compared with the performance of several traditional detection methods.^32^ Moreover Sa et al.^33^ proposed Faster‐RCNN with small annotated clinical datasets in their study, and they used 974 training images and tested 108 images based on their proposed network. Their network can achieve much better performance compared to the traditional sliding window detection method on handcrafted features using only a small annotated clinical dataset.

Image segmentation is used extensively in the field of medical imaging. First, image segmentation needs intensive annotation, that is, to label every pixel of the focus area, which reduces the requirements of the network on the amount of image data. Second, image segmentation can clearly show an abnormal area in the image, which is a.dvantageous in the interpretability of the network.^16^, ^34^ However, in the MRI image of the cervical spine, the diseased area is irregular and the boundary is not clear, which makes it difficult to segment and label the image. Consequently, generating an accurate image segmentation label is difficult. This in turn will have a substantial impact on the network performance. Therefore, we recommend using detection networks to solve this problem rather than the U‐net architecture.

Many studies have proposed boosting classifier for improvement of object‐detection tasks in many categories. The idea of boosting is to modify the final result by fusing the results of multiple models.^35^ For example, in the classification of the ImageNet dataset, if the classification result of four models for an image is “goldfinch,” and that of one model is “house finch,” after boosting, the final result will be output as “goldfinch.” Among them, the five models are independent and can have different results, such as model 1 is ResNet and model 2 is DenseNet.^36^ In this paper, the synergic classifier image is combined, thereby causing the classification results of the three sub‐networks to exhibit a higher consistency. Next, we use the idea of boosting to suppress the results of the three sub‐networks again, which we think is another advantage of the model. In our study, the detection of cervical lesions is characterized such that each image only is classified as either normal or as a certain disease, and there is only one lesion area, which guarantees the feasibility of adding synergic classifiers to the network. However, typically, in natural image target detection, this method is not suitable because it deals with multiple classifications. By adding a synergic classifier in this study, the feature extractor of the three sub‐networks shows consistency for the classification information of images. The number of parameters of the synergic classifier is increased by 18 K, which is approximately 2% of a single sub‐network. Therefore, although the number of parameters of the whole network increases by a factor of three, the number of parameters of a single sub‐network does not increase much. Therefore, there is no requirement for additional data to overfit the network because of the large size of the network parameters.

Our study has some limitations. First, currently, the deep learning of object‐detection task is updated very quickly, and the method we adopted may have a lag compared to the latest method. Second, the dataset used in the study is limited to our hospital imaging system. Although this method can ensure the uniformity of data, it also results in an insufficient data volume compared with other databases, as well as poor scalability. If the dataset comes from multiple hospitals, it would greatly improve the persuasiveness and practicality of the experiment.

## 5 CONCLUSION

In this study, we implemented a Faster R‐CNN combined with a backbone convolutional feature extractor using ResNet‐50 and the VGG‐16 network to detect lesions on cervical MRI images. Experimental results showed that Faster R‐CNN improves the possibility of diagnosing lesions from cervical MRI. Indirectly, our study showed that deep learning can help to detect cervical diseases on MRI, which is a value addition to the field. In the future, we hope that more evidence‐based datasets can be established with the intentions of providing a more reproducible approach to analyze MRI incorporated into deep learning and designing the MRI's criterion for assessing diseases. Furthermore, it can be based on a deep learning network to build a normalized analysis for clinical research studies of MRI. Furthermore, the technique of Faster R‐CNN can provide the possibility of auxiliary‐diagnosing for radiologists and spine surgeons in the field of spinal injury.

## CONFLICT OF INTEREST

All authors declared that there was no conflict of interests involved in this study.
